# Genetic variants of *SP‐D* confer susceptibility to radiation pneumonitis in lung cancer patients undergoing thoracic radiation therapy

**DOI:** 10.1002/cam4.2088

**Published:** 2019-03-21

**Authors:** Li Xu, Junhong Jiang, Yunming Li, Ling Zhang, Zhihui Li, Jing Xian, Chaoyang Jiang, Yong Diao, Xiaomei Su, Hongyu Xu, Yue Zhang, Tao Zhang, Zhenzhou Yang, Bangxian Tan, Hua Li

**Affiliations:** ^1^ Cancer Center The General Hospital of Western Theater Command Chengdu China; ^2^ Department of Nutrition and Dietetics Affiliated Hospital of North Sichuan Medical College Nanchong China; ^3^ Department of Oncology The First People’s Hospital of Neijiang Neijiang China; ^4^ Department of Statistics The General Hospital of Western Theater Command Chengdu China; ^5^ Department of Statistics, College of Mathematics Southwest Jiaotong University Chengdu China; ^6^ Cancer Center The Second affiliated Hospital of Chongqing Medical University Chongqing China; ^7^ Department of Oncology Affiliated Hospital of North Sichuan Medical College Nanchong China

**Keywords:** genetic polymorphisms, lung cancer, radiation pneumonitis, SP‐D, susceptibility gene

## Abstract

**Background:**

Surfactant protein D (SP‐D) is an innate immunity molecule in the alveoli. However, the associations between genetic variants of *SP‐D *and radiation pneumonitis (RP) have never been investigated.

**Methods:**

The Linkage disequilibrium of *SP‐D *and tagSNPs were analyzed by using Haploview 4.1. Eight tagSNPs were genotyped among 396 lung cancer patients who received thoracic radiation therapy with follow–up time (median [P25, P75]: 11[6, 18]) using improved multiplex ligation detection reaction (iMLDR). The associations between clinical characteristics, tagSNP alleles, genotypes, haplotypes and onset time of grade ≥2 or ≥3 RP were evaluated by using univariate and multivariate Cox proportional hazard regression model.

**Results:**

Three tagSNPs of *SP‐D *(rs1998374, rs911887 and rs2255326) were significantly associated with grade ≥2 RP in multivariate analysis with multiple testing (Q test). The rs199874 had a protective effect for grade ≥2 RP in the dominant model (Hazard ratio (HR), 0.575; 95% confidence interval (CI), 0.378‐0.875). The homozygous mutant genotype for rs911887 had risk effect for grade ≥2 RP (HR, 2.209; 95% CI, 1.251‐3.902). The A mutant allele of rs2255326 also showed an elevated risk for grade ≥2 RP (HR, 1.777; 95% CI, 1.283‐2.461) and this risk effect was still significant in the recessive genetic model (HR, 3.320; 95% CI, 1.659‐6.644) and dominant genetic model (HR, 1.773; 95% CI, 1.166‐2.696). Compared to the lung cancer patients bearing the most common haplotype C‐G‐T, the patients bearing the haplotype T‐A‐C (rs1998374‐rs2255326‐rs911887) showed a significant risk of both grade ≥2 RP (HR, 1.885; 95% CI, 1.284‐2.765) and grade ≥3 RP (HR, 2.256; 95% CI, 1.248‐4.080).

**Conclusions:**

Genetic variants of *SP‐D* were associated with risk of RP development in lung cancer patients receiving thoracic radiotherapy.

## 1 INTRODUCTION

With the increasing lung cancer patients diagnosed, more and more lung cancer patients receive thoracic radiotherapy as a part of standard treatment regimen.^1^ However, approximately 16%‐30% of lung cancer patients experience moderate to severe radiation‐induced pneumonitis (RP) within the first 3 months of thoracic radiation therapy, even for those who were treated with intensity–modulated radiation therapy (IMRT).^2^ RP limits the escalation of radiation dose necessary to achieve curative effects while reducing the quality of life for patients.^3^, ^4^, ^5^ Therefore, establishing reliable predictors of RP is a critical step towards maximizing efficacy of treatment for lung cancer patients while minimizing adverse effects associated with thoracic radiation.

Multiple therapeutic and patient–related RP risk factors include performance status (PS), smoking status, chemotherapy, and dosimetric parameters.^6^ However, they are not sufficient to fully explain why under similar radiotherapy doses fraction some patients develop RP while the rest never do, which suggests a genetic basis for RP. RP genetic association studies had identified some RP susceptibility genes, further supporting a genetic basis in RP development. These RP susceptibility genes identified thus far were involved in the DNA repair pathways,^7^, ^8^, ^9^ oxidative stress pathways,^10^ cellular signaling pathways,^11^ and inflammatory response to ionizing radiation.^12^, ^13^, ^14^

Surfactant protein D (SP‐D), a component of lung surfactants, which reduces surface tension at the pulmonary air–liquid interface^15^, ^16^, ^17^ and enhances defense against pathogens as the first line of innate pulmonary immunity,^18^, ^19^, ^20^, ^21^ has never been investigated with regard to the association between its genetic variants and the risk of RP. Previous study showed that serum levels of *SP‐D *in RP patients were elevated.^22^, ^23^, ^24^ More specifically, the genetic polymorphisms in *SP‐D* were associated with some lung injuries such as COPD,^25^ interstitial pneumonia,^26^ asthma,^27^ lung cancer,^26^ and infectious lung diseases.^28^, ^29^ Therefore, we suspected that single–nucleotide polymorphisms (SNPs) of *SP‐D* would also be associated with RP.

In this study, two endpoints of grade ≥2 or grade ≥3 RP were observed and the potential associations of *SP‐D* were explored in lung cancer patients treated with thoracic radiotherapy. To comprehensively investigate this association, the alleles, genotypes and haplotypes of tagSNPs in *SP‐D *were analyzed after clinical–risk RP factors were evaluated.

## 2 MATERIALS AND METHODS

### 2.1 Patients

Lung cancer patients receiving thoracic radiotherapy were recruited at the Department of Radiation Oncology at Daping Hospital (Chongqing, China) between February 2006 and March 2011, and were continually recruited at the Cancer Center of Chengdu Military General Hospital (Chengdu, China) between April 2011 and April 2017. The inclusion criteria were (a) histological and cytological confirmation of lung cancer; (b) genomic DNA samples and clinical data availability; (c) patients who did not develop RP during the follow–up period must have received a radiation dose ≥40 Gy; (d) patient records with at least a 6‐month follow–up period. The exclusion criteria included (a) patients who had previous thoracic irradiation; (b) patients who had severe cardiopulmonary diseases; (c) patients who had blood relatives already enrolled in the study. Written informed consent was obtained from every patient before radiotherapy treatment. Key patient characteristics, including PS, smoking history, lung cancer stage, and chemotherapy, were all recorded. A 1‐ml peripheral blood sample was collected from each patient before radiotherapy, and genomic DNA was extracted from the blood samples using Wizard Genomic DNA Purification Kit (Promega, USA). The isolated DNA samples’ purity and concentration were determined by spectrophotometric measurement of absorbances at 260 and 280 nm.

### 2.2 Treatment and follow‐up

All patients received radiotherapy with 6‐MV X‐rays from a linear accelerator (Varian, USA). The dosimetric parameters were obtained from the treatment planning system. The median total radiation dose was 55.1 Gy (range: 22 to 72 Gy), with a mean of 2.2 Gy (range: 1.5 to 3 Gy) administered per radiation treatment. 96.2% of the patients (n = 381) received IMRT (intensity–modulated radiation therapy) and others received three–dimensional conformal radiotherapy with total radiation dose ranging from 40 Gy to 70 Gy. The dosimetric parameters for the risk of RP such as the percentage of lung volume receiving greater than 5, 10, 20, 30 Gy (V5, V10, V20, V30) were obtained from the dose–volume histograms (DVH).

During radiation therapy, patients were monitored weekly. They were checked every month from 1 to 3 months after radiotherapy and then every 3 months afterwards during follow‐up observations. Follow‐up evaluations included interval history, physical examination, chest CT or PET/CT, pulmonary functional tests, and routine blood tests. RP was assessed firstly at each follow‐up visit and diagnosed by clinical presentation and radiographic abnormalities, including ground–glass opacity, attenuation, or consolidation changes within the radiation field. Then, RP was graded by two radiation oncologists independently who were blinded from the genotyping results according to the Common Terminology Criteria for Adverse Events version 3.0. Patients with clinical symptoms such as shortness of breath, dry cough, low–grade fever, chest tightness and/or pain were graded >2 RP. The RP patients with oxygen therapy in medical history were graded >3 RP. The time to end–point development was calculated from the beginning of the radiation therapy for each patient to the final follow–up date October, 2017.

### 2.3 TagSNPs selection and genotyping

Linkage disequilibrium of *SP‐D* and tagSNPs in *SP‐D* were analyzed by using HaploView 4.0 software (Supplementary Figure S1). A total of eight tagSNPs (rs721917, rs2243639, rs726288, rs1923536, rs1998374, rs911887, rs2255326 and rs75074551), which captured 100% of 36 alleles with MAF >0.1 with a mean r^2^ of 0.959, were genotyped by experts who were blinded to the clinical information using improved multiplex ligation detection reaction (iMLDR) method (Genesky Biotechnologies Inc, Shanghai, China). A 5% blind, random sample of study subjects was genotyped twice and the genotype concordance rate was 100% and the call rate was 100%.

### 2.4 Statistical methods

The Hardy‐Weinberg equilibrium (HWE) test was conducted via Pearson χ^2^ goodness‐of‐fit test. We observed two endpoints: development of grade ≥2 RP and development of grade ≥3 RP. The time to the end‐point was calculated from the start of radiotherapy for each patient. The SPSS 16.0 statistical package (SPSS, Chicago, IL) was used for the statistical analyses. The associations between clinical characteristics, tagSNP alleles, genotypes, haplotypes and onset time of grade ≥2 or ≥3 RP were evaluated by using univariate and multivariate Cox proportional hazard regression model. Because eight SNPs and many tests were performed, the Q value that represents a measure of significance in terms of the false discovery rate was used to adjust the significance level for individual SNPs.^30^ Q value was calculated by the Q value package implemented in the R software. Kaplan‐Meier curve and log–rank test were used to assess the differences of overall RP probability. For the positive tagSNPs, a dominant genetic model was considered. Estimation of haplotype frequencies was completed via PHASE v2.1.1 software (http://stephenslab.uchicago.edu/software.html#phase). All *P*‐values refer to two–sided tests, with *P* < 0.05 considered as statistically significant.

## 3 RESULTS

### 3.1 Patients, treatment, and radiation dosimetric characteristics

A total of 396 patients with a mean age of 59.15 years (range, 23 to 80 years) were analyzed in this study (Table 1). Most of them (88.13%, n = 349) had stage III/IV lung cancer (according to the 7th lung cancer TNM classification and staging system) and 97.47% (n = 386) were treated with a combination of chemotherapy and radiotherapy. The chemotherapies of these patients were irinotecan and platinum for SCLC. Pemetrexed and cisplatin were adopted as first–line chemotherapy for adenocarcinoma in NSCLC and paclitaxel and cisplatin were used for squamous cell carcinoma and adenosquamous carcinoma of the lung (ASC). Dosimetric parameters for each subvolume V5, V10, V20 and V30 were shown in Table 1. The median follow–up periods were 11.4 months (range: 6‐58).

**Table 1:** Characteristics (n = 396) of lung cancer patients treated with thoracic radiotherapy

	Descriptive statistics
Gender, n (%)	
Male	309 (78.03)
Female	87 (21.97)
Age (y), mean ± SD	59.15 ± 9.72
Age group (y), n (%)	
≤60	205 (51.77)
>60	191 (48.23)
Histology, n (%)	
SCLC	97 (24.49)
NSCLC	299 (75.51)
Adenocarcinoma	116 (38.80)
Squamous cell carcinoma	173 (57.86)
Adenosquamous carcinoma	10 (3.34)
Tumor location in the lung, n (%)	
Upper lobe	176 (44.44)
Middle lower lobes	220 (55.56)
Stage, n (%)	
Ⅰ, Ⅱ	47 (11.87)
ⅢA	99 (25.00)
ⅢB	102 (25.76)
Ⅳ	148 (37.37)
PS, n (%)	
≤2	383 (96.72)
>2	13 (3.28)
Smoking history, n (%)	
Never	155 (39.14)
Ever	241 (60.86)
Pulmonary lobectomy, n (%)	
No	332 (83.84)
Yes	64 (16.16)
Chemotherapy, n (%)	
No	10 (2.53)
Irinotecan and platinum	97 (24.49)
Pemetrexed and cisplatin	111 (28.03)
Paclitaxel and cisplatin	178 (44.95)
Radiation dose fractionation (Gy)	
Mean ± SD	2.19 ± 0.58
Median (P_25_, P_75_)	2 (2,2.2)
Radiation dose fractionation group (Gy), n (%)	
≤2 (conventional radiotherapy)	276 (69.70)
>2 (hypofractionation)	120 (30.30)
Radiation dose (Gy)	
Mean ± SD	55.09 ± 7.12
Median (P_25_, P_75_)	56 (50,60)
V5 (%)	
Mean ± SD	51.22 ± 15.64
Median (P_25_, P_75_)	51.15 (39.2,60.9)
V10 (%)	
Mean ± SD	35.75 ± 12.31
Median (P_25_, P_75_)	35.82 (26.73,44.13)
V20 (%)	
Mean ± SD	20.54 ± 7.99
Median (P_25_, P_75_)	20 (14.9,26)
V30 (%)	
Mean ± SD	11.97 ± 5.85
Median (P_25_, P_75_)	12 (8,16)

NSCLC, nonsmall cell carcinoma; PS, performance status of ECOG score standard; SCLC, small cell carcinoma; V10 (%), percentage of the lung volume that received more than 10 Gy; V20 (%), percentage of the lung volume that received more than 20 Gy; V30 (%), percentage of the lung volume that received more than 30 Gy; V5 (%), percentage of the lung volume that received more than 5 Gy.

### 3.2 Clinical and dosimetric variables associated with grade ≥2 or ≥3 RP

After radiotherapy, 88 (22.22%) patients experienced grade ≥2 RP and 38 (9.60%) patients developed grade ≥3 RP. The mean occurrence time of grade ≥2 RP or grade ≥3 RP were 2.8 months and 2.7 months, respectively. To determine whether any confounding factors were influencing the risk of RP, the association between RP and clinical–dosimetric characteristics was investigated first. The clinical variables associated with grade ≥2 RP were PS, smoking history, radiation dose and V5~V30 (Table 2). The clinical variables associated with grade ≥3 RP were age, PS, radiation dose and V5~V30 in univariate analysis (Table 2). In the stepwise multivariate Cox proportional hazard regression model, V30 was associated with grade ≥2 RP (HR, 1.063; 95% CI, 1.026‐1.101) and both age group (HR, 2.373; 95% CI, 1.197‐4.703) and V10 (HR, 1.042; 95% CI, 1.016‐1.070) were associated with grade ≥3 RP (Table 3).

**Table 2:** Univariate analysis between clinical characteristics and grade ≥2 RP or grade ≥3 RP (n = 396)

	Grade ≥2 RP			Grade ≥3 RP		
	HR	95% CI	*P*	HR	95% CI	*P*
Gender						
Male	1			1		
Female	0.706	0.405‐1.230	0.219	0.791	0.348‐1.797	0.576
Age group (y)						
≤60	1			1		
>60	1.277	0.840‐1.942	0.252	2.351	1.186‐4.659	0.014
Histology						
SCLC	1			1		
Adenocarcinoma	0.900	0.508‐1.595	0.719	0.760	0.323‐1.791	0.531
Squamous cell carcinoma	0.930	0.554‐1.561	0.785	0.764	0.351‐1.663	0.497
Adenosquamous carcinoma	1.440	0.432‐4.798	0.552	1.968	0.436‐8.880	0.379
Tumor location in the lung						
Upper lobe	1			1		
Middle lower lobes	0.936	0.615‐1.425	0.759	0.642	0.339‐1.217	0.174
Stage						
Ⅰ,Ⅱ	1			1		
ⅢA	1.581	0.716‐3.492	0.257	1.435	0.290‐7.109	0.658
ⅢB	1.452	0.652‐3.231	0.361	3.590	0.821‐15.698	0.090
Ⅳ	1.227	0.562‐2.676	0.607	2.475	0.566‐10.824	0.229
PS						
≤2	1			1		
>2	2.370	1.034‐5.431	0.041	3.584	1.271‐10.106	0.016
Smoking history						
Never	1			1		
Ever	1.617	1.023‐2.556	0.040	1.422	0.717‐2.818	0.313
Pulmonary lobectomy						
No	1			1		
Yes	0.805	0.438‐1.480	0.485	0.979	0.409‐2.340	0.961
Chemotherapy						
No	1			1		
Irinotecan and platinum	1.097	0.259‐4.654	0.900	0.516	0.114‐2.326	0.389
Pemetrexed and cisplatin	0.987	0.233‐4.188	0.986	0.367	0.079‐1.698	0.199
Paclitaxel and cisplatin	1.049	0.253‐4.341	0.947	0.410	0.094‐1.783	0.234
Radiation dose fractionation group (Gy)						
≤2 (conventional radiotherapy)	1			1		
>2 (hypofractionation)	0.847	0.530‐1.355	0.489	0.509	0.224‐1.156	0.107
Radiation dose (Gy)	0.971	0.945‐0.997	0.032	0.951	0.916‐0.987	0.009
V5 (%)	1.017	1.003‐1.030	0.016	1.024	1.003‐1.045	0.024
V10 (%)	1.028	1.011‐1.046	0.001	1.042	1.015‐1.069	0.002
V15 (%)	1.038	1.012‐1.065	0.004	1.058	1.018‐1.100	0.004
V30 (%)	1.063	1.026‐1.101	<0.001	1.063	1.008‐1.121	0.023

*P* were calculated by univariate Cox proportional hazard regression model.

95% CI, 95% confidence interval; HR, hazard ratio; PS, performance status of ECOG score standard; RP, Radiation Pneumonitis; SCLC, small cell carcinoma; V10 (%), percentage of the lung volume that received more than 10 Gy; V20 (%), percentage of the lung volume that received more than 20 Gy; V30 (%), percentage of the lung volume that received more than 30 Gy; V5 (%), percentage of the lung volume that received more than 5 Gy.

**Table 3:** Multivariate analysis between clinical characteristics and grade ≥2 RP or grade ≥3 RP (n = 396)

	Grade ≥2 RP			Grade ≥3 RP		
	HR	95% CI	*P*	HR	95% CI	*P*
Age group (y)						
≤60				1		
>60				2.373	1.197‐4.703	0.013
V10 (%)				1.042	1.016‐1.070	0.002
V30 (%)	1.063	1.026‐1.101	<0.001			

Stepwise multivariate Cox proportional hazard regression model was used to select independent risk factors and the probabilities of entry and removal were 0.05 and 0.10.

95% CI, 95% confidence interval; HR, hazard ratio; RP, Radiation Pneumonitis; V10 (%), percentage of the lung volume that received more than 10 Gy; V30 (%), percentage of the lung volume that received more than 30 Gy.

### 3.3 Univariate and multivariate analysis of tagSNPs association with grade ≥2 RP or grade ≥3 RP

The results of Pearson χ² goodnessof‐fit test showed that allele and genotype frequencies of all tagSNPs were in Hardy‐Weinbergequilibrium (P > 0.05) in both the patient and the control populations, indicating our samples were a random mating population without selection. With respect to grade ≥2 RP, the associations were statistically significant for rs1998374, rs911887 and rs2255326 in multivariate analysis after adjusting confounding factors and with correction with Q value^30^ (Table 4). The C mutant allele of rs1998374 was protective against grade ≥2 RP in dominant genetic model (HR, 0.575; 95% CI, 0.378‐0.875) whereas the homozygous genotype of rs911887 increased the risk of grade ≥2 RP in the lung cancer patients (HR, 2.209; 95% CI, 1.251‐3.902). For rs2255326, the mutant allele A was associated with an increased risk of grade ≥2 RP (HR, 1.777; 95% CI, 1.283‐2.461) and these significant associations were still seen in both dominant (HR, 1.773; 95% CI, 1.166‐2.696) and recessive models (HR, 3.320; 95% CI, 1. 659‐6.644). Although these associations were detected between grade ≥3 RP and rs1998374 or rs2255326 in multivariate analysis, they could not reach significance level after corrected with Q value (Table 4).

**Table 4:** Associations between tagSNP alleles, genotypes of *SP‐D *and grade ≥2 RP or grade ≥3 RP

tagSNP allele/genotypes	All Radio‐therapy patients	Grade ≥2 RP									Grade ≥3 RP								
		case No.	Univariate analysis^a^				Multivariate analysis^b^				Case No.	Univariate analysis^a^				Multivariate analysis^c^			
			HR	95% CI	*P*	*Q* d	HR	95% CI	*P*	*Q* d		HR	95% CI	*P*	*Q* d	HR	95% CI	*P*	*Q* d
rs721917																			
G	477	110	1				1				46	1				1			
A	315	66	0.909	0.670‐1.234	0.542	0.587	0.924	0.681‐1.253	0.610	0.670	30	0.987	0.623‐1.563	0.955	0.962	0.928	0.585‐1.472	0.750	0.974
GG	143	36	1				1				15	1				1			
GA	191	38	0.781	0.495‐1.232	0.288	0.416	0.813	0.515‐1.282	0.372	0.484	16	0.791	0.391‐1.599	0.513	0.785	0.714	0.351‐1.453	0.352	0.654
AA	62	14	0.905	0.488‐1.677	0.750	0.750	0.921	0.497‐1.709	0.795	0.827	7	1.080	0.44‐2.649	0.867	0.962	0.965	0.391‐2.381	0.938	0.974
rs2243639																			
C	612	140	1				1				59	1				1			
T	180	36	0.874	0.606‐1.261	0.471	0.532	0.911	0.631‐1.315	0.618	0.670	17	0.984	0.574‐1.687	0.952	0.962	0.991	0.576‐1.704	0.974	0.974
CC	227	54	1				1				22	1				1			
CT	158	32	0.848	0.548‐1.314	0.461	0.532	0.886	0.572‐1.373	0.588	0.670	15	0.983	0.51‐1.895	0.959	0.962	0.963	0.497‐1.865	0.910	0.974
TT	11	2	0.775	0.189‐3.181	0.724	0.750	0.875	0.213‐3.602	0.854	0.854	1	0.953	0.128‐7.073	0.962	0.962	1.151	0.152‐8.701	0.891	0.974
rs726288																			
C	616	144	1				1				62	1				1			
T	176	32	0.758	0.517‐1.111	0.156	0.267	0.757	0.516‐1.11	0.154	0.287	14	0.793	0.444‐1.416	0.432	0.750	0.926	0.516‐1.663	0.798	0.974
CC	237	58	1				1				26	1				1			
CT	142	28	0.771	0.491‐1.211	0.259	0.397	0.791	0.503‐1.242	0.308	0.435	10	0.635	0.306‐1.316	0.222	0.412	0.728	0.349‐1.518	0.397	0.666
TT	17	2	0.478	0.117‐1.956	0.304	0.416	0.443	0.108‐1.814	0.258	0.394	2	1.128	0.268‐4.752	0.870	0.962	1.809	0.408‐8.022	0.435	0.666
rs1923536																			
C	753	163	1				1				71	1				1			
T	39	13	1.548	0.880‐2.724	0.129	0.259	1.540	0.875‐2.709	0.134	0.269	5	1.375	0.555‐3.406	0.491	0.785	1.379	0.557‐3.416	0.487	0.667
CC	358	76	1				1				34	1				1			
CT	37	11	1.376	0.731‐2.590	0.322	0.419	1.380	0.733‐2.597	0.318	0.435	3	0.842	0.259‐2.741	0.775	0.962	0.908	0.278‐2.962	0.873	0.974
TT	1	1	8.091	1.115‐58.687	0.039	0.112	6.388	0.878‐46.459	0.067	0.161	1	20.259	2.706‐151.679	0.003	0.040	6.030	0.745‐48.816	0.092	0.240
rs1998374																			
T	460	116	1				1				54	1				1			
C	332	60	0.696	0.51‐0.951	0.023	0.074	0.695	0.509‐0.95	0.022	0.072	22	0.557	0.339‐0.915	0.021	0.077	0.587	0.357‐0.965	0.036	0.176
TT	133	40	1				1				21	1				1			
CT	194	36	0.582	0.371‐0.913	0.018	0.068	0.584	0.372‐0.916	0.019	0.072	12	0.377	0.186‐0.767	0.007	0.046	0.380	0.187‐0.774	0.008	0.074
CC	69	12	0.551	0.289‐1.051	0.070	0.183	0.549	0.288‐1.046	0.068	0.161	5	0.451	0.17‐1.196	0.109	0.219	0.510	0.19‐1.372	0.183	0.365
CT+CC	263	48	0.574	0.377‐0.873	0.009	0.041	0.575	0.378‐0.875	0.008	0.040	17	0.396	0.209‐0.751	0.005	0.040	0.410	0.216‐0.781	0.007	0.074
rs911887																			
T	514	98	1				1				41	1				1			
C	278	78	1.518	1.127‐2.044	0.006	0.039	1.457	1.082‐1.963	0.013	0.057	35	1.596	1.016‐2.505	0.042	0.118	1.513	0.963‐2.377	0.072	0.217
TT	168	30	1				1				10	1				1			
CT	178	38	1.203	0.745‐1.941	0.450	0.532	1.183	0.733‐1.91	0.491	0.608	21	2.001	0.942‐4.249	0.071	0.162	1.848	0.868‐3.933	0.111	0.241
CC	50	20	2.436	1.383‐4.29	0.002	0.018	2.209	1.251‐3.902	0.006	0.040	7	2.407	0.916‐6.324	0.075	0.162	2.220	0.844‐5.839	0.106	0.241
CT+CC	228	58	1.457	0.938‐2.264	0.094	0.204	1.406	0.904‐2.187	0.130	0.269	28	2.089	1.015‐4.301	0.046	0.118	1.929	0.935‐3.98	0.075	0.217
rs2255326																			
G	642	125	1				1				52	1				1			
A	150	51	1.817	1.312‐2.516	<0.001	0.013	1.777	1.283‐2.461	0.001	0.009	24	2.017	1.243‐3.271	0.004	0.040	1.914	1.18‐3.105	0.009	0.074
GG	262	47	1				1				18	1				1			
GA	118	31	1.487	0.945‐2.34	0.086	0.204	1.540	0.978‐2.425	0.063	0.161	16	2.015	1.027‐3.951	0.042	0.118	2.029	1.031‐3.993	0.041	0.176
AA	16	10	4.058	2.048‐8.041	<0.001	0.013	3.320	1.659‐6.644	0.001	0.009	4	3.787	1.281‐11.194	0.016	0.069	2.975	0.999‐8.862	0.050	0.187
GA+AA	134	41	1.758	1.156‐2.673	0.008	0.041	1.773	1.166‐2.696	0.007	0.040	20	2.222	1.176‐4.202	0.014	0.069	2.171	1.146‐4.11	0.017	0.113
rs75074551																			
G	740	169	1				1				70	1				1			
A	52	7	0.570	0.268‐1.214	0.145	0.267	0.615	0.289‐1.311	0.208	0.360	6	1.225	0.532‐2.82	0.633	0.866	1.364	0.592‐3.147	0.466	0.667
GG	345	81	1				1				32	1				1			
GA	50	7	0.578	0.267‐1.251	0.164	0.267	0.620	0.286‐1.343	0.225	0.366	6	1.303	0.545‐3.117	0.551	0.796	1.423	0.593‐3.418	0.430	0.666
AA	1																		

95% CI, 95% confidence interval; HR, hazard ratio; tagSNP, tag single–nucleotide polymorphisms.

a Univariate analysis adopted Cox proportional hazard regression model with adjustment for no factor.

b Multivariate analysis adopted Cox regression model with adjustment for V30 (%).

c Multivariate analysis adopted Cox regression model with adjustment for age group and V10 (%).

d *Q* was the result of multiple comparison adjustment by Q test.

### 3.4 SNPs and cumulative probability of grade ≥2 or ≥ 3 RP

**Figure 1:**
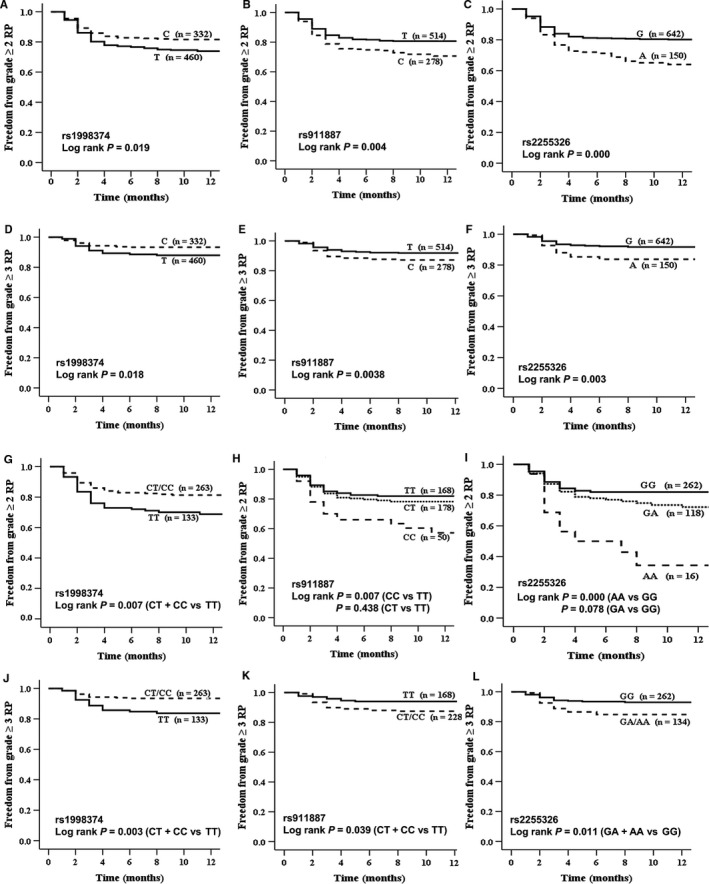
Kaplan‐Meier curve and log–rank test comparing the cumulative RP‐free probability of time to an adverse outcome between patients with alleles and genotypes of rs1998374, rs911887 and rs2255326. K‐M allele analysis of rs1998374, rs911887 and rs2255326 for grade ≥2 RP were (A‐C) and for grade ≥3 RP were (D‐F) respectively. K‐M genotype analysis of rs1998374, rs911887 and rs2255326 for grade ≥2 RP were (G‐I) and for grade ≥3 RP were (J‐L), respectively

The overall probabilities of grade ≥2 or grade ≥3 RP were assessed with the Kaplan‐Meier curve and log–rank test (Figure1). Patients bearing mutant allele C of rs1998374 showed lower risk for grade ≥2 RP and grade ≥3 RP compared to wild–type allele T. This protect effect was also seen in dominant model (Figure 1A,D). Patients with mutant allele of rs911887 (Figure 1B,E) or of rs2255326 (Figure 1C,F) had significantly higher risk for both grade ≥2 RP and grade ≥3 RP compared to wild–type allele. This risk effect was also seen in homozygous mutant genotype compared to wild–type genotype for grade ≥2 RP (Figure 1H,I) and in dominant model for grade ≥3 RP (Figure 1K,L).

### 3.5 Haplotype association analysis

To further investigate the combined effect of the 3 tagSNPs (rs1998374, rs911887, and rs2255326) on the risk of RP, we estimated the frequencies of each haplotype by PHASE software and performed haplotype analysis using Cox proportional hazard regression model. There were only 4 haplotypes with over 10% frequency among eight haplotypes in the patients, other rare haplotypes were combined together as “others” in our statistical analysis (Table 5). The T‐A‐C haplotype was significantly associated with increased risk for both grade ≥2 RP and grade ≥3 RP when compared the most common haplotype (C‐G‐T). Lung cancer patients bearing the T‐A‐C haplotype showed approximately twofold risk of RP than those bearing the C‐G‐T haplotype for both grade ≥2 RP (HR, 1.885; 95% CI, 1.284‐2.765) and grade ≥3 RP (HR, 2.256; 95% CI, 1.248‐4.080).

**Table 5:** Haplotype (rs1998374‐rs2255326‐rs911887) frequency estimation and haplotype association with grade ≥2 RP or grade ≥3 RP

Haplotype	All Radio‐Therapy patients	Grade ≥2 RP							Grade ≥3 RP						
		Case No.	Univariate analysis^a^			Multivariate analysis^b^			Case No.	Univariate analysis a			Multivariate analysis^c^		
			HR	95% CI	*P*	HR	95% CI	*P*		HR	95% CI	*P*	HR	95% CI	*P*
C‐G‐T	327	59	1			1			22	1			1		
T‐G‐T	180	35	1.093	0.719‐1.660	0.678	1.128	0.742‐1.714	0.572	17	1.417	0.753‐2.669	0.280	1.407	0.744‐2.661	0.293
T‐A‐C	143	47	1.916	1.306‐2.811	<0.001	1.885	1.284‐2.765	0.001	22	2.345	1.299‐4.235	0.005	2.256	1.248‐4.080	0.007
T‐G‐C	130	30	1.311	0.845‐2.034	0.227	1.267	0.816‐1.967	0.292	13	1.493	0.752‐2.964	0.252	1.357	0.682‐2.698	0.384
other	12	5	2.221	0.891‐5.535	0.087	1.781	0.711‐4.458	0.218	2	2.447	0.575‐10.407	0.226	1.592	0.372‐6.811	0.531

Haplotype: rs1998374‐rs2255326‐rs911887, others included haplotype C‐A‐T, C‐A‐C, T‐A‐T, C‐G‐C with below 0.03 frequency.

a Univariate analysis adopted Cox proportional hazard regression model with adjustment for no factor.

b Multivariate analysis adopted Cox regression model with adjustment for V30 (%).

c Multivariate analysis adopted Cox regression model with adjustment for age group and V10 (%).

## 4 DISCUSSION

In the present study, three tagSNPs (rs1998374, rs911887, rs2255326) were significantly associated with grade ≥2 RP and haplotype (T‐A‐C) in *SP‐D* was significantly associated with both grade ≥2 RP and grade ≥3 RP in lung cancer patients treated with radiotherapy. To our knowledge, this is the first report on an association between *SP‐D* and RP. It is worth noting that the eight tagSNPs tested captured 100% of 36 alleles in *SP‐D* with mean r^2^ of 0.959, which means 36 SNPs in *SP‐D* were investigated in this study.

RP, a complication after radiotherapy, is involved clinical characteristics, therapy–related factors and follow–up tasks. Therefore, the design and measures of RP genetic association study would be more complex than common association studies of disease.^31^, ^32^ One critical issue is the confounders. In our study, nongenetic factors including clinical information and radiotherapy–related factors were comprehensively considered before investigation of novel genetic risk factors. In addition to nongenetic factors usually analyzed in other RP genetic association studies,^7^, ^9^, ^11^, ^33^, ^34^ tumor location and more precise dosimetric parameters such as V5, V10 and V20 mentioned in some clinic studies as prediction factors for RP^5^, ^35^, ^36^, ^37^ were also analyzed in this study. We found that PS, smoking status, age and dosimetric parameters were significantly associated with RP. Whether chemotherapy was performed or not was regarded as an influencing factor of RP in some studies,^38^ but in our study only 2.5% patients received radiotherapy alone and the results did not reach statistical significance (*P *_grade ≥2 _= 0.841 and *P*_grade ≥3_ = 0.097). Further analysis about different chemotherapy drugs used among patients showed no association with RP risk. Another critical issue is the stringency of RP diagnosis criteria. First, to ensure case group are strictly defined, RP diagnosis and grading should be defined properly and accurately recorded. In our study, 2 radiation oncologists diagnosed and graded RP independently and were blinded to the genetic information. Second, our follow–up schedule was considered to record RP occurrence accurately. Lastly, to reduce the possibility of false non‐RP phenotypes in the control group, the follow–up period in our study was extended to a median of 11.4 months, longer than normal occurrence time of RP (<6 months).^6^

The allele and genotype analysis in this study consistently revealed that the genetic variants of *SP‐D *were associated with RP. The mutant alleles of rs911887 and rs2255326 were risk factors for RP while the mutant allele of rs1998374 was a protect factor for RP. Their different effect for RP roused our interest to further analyze their combined effect for RP using haplotype analysis. Lung cancer patients treated with radiotherapy bearing C‐G‐T haplotype had about twofold higher risk of RP than those bearing T‐A‐C haplotype, strongly suggesting that the genetic variants of *SP‐D* could be a genetic biomarker in predicting RP development among lung cancer patients.

In our cohort of 396 samples, there was only one patient with a homozygous mutant genotype TT of rs1923536. Although this patient developed high–grade RP, the association between rs1923536 and RP must be reinvestigated in a large sample. Therefore, this tagSNP was excluded from haplotype analysis.

Since RP susceptibility loci could be predictors before radiotherapy, screening RP susceptibility genes has attracted researchers’ interest lately. However, maybe for the reason that complex RP follow–up tasks limited the sample size, to explore RP susceptibility loci usually used not genome–wide association study (GWAS) but only candidate gene approach. Until now, RP susceptibility loci were identified in DNA repair–related, inflammation–related, angiogenesis–related and stress response–related pathways with different underlying mechanisms.^32^ SP‐D, a surfactant proteins known for its contribution to the host's lung immunity,^39^ has been mentioned to be biomarker of severe RP after radiotherapy.^40^ This study expanded the range of RP candidate genes to a new gene encoded pulmonary surfactant protein D, suggesting another mechanism may underlying the pathogenesis of RP. To further determine the effects of *SP‐D* SNPs and its role in RP, the functional identification of SNPs linked to variable expression levels of SP‐D and more biological studies would be performed later.

Although our patients were recruited from two hospitals, studies with larger sample size or more patients from multi‐center studies are needed to validate our findings.

Despite its limitations, this study is the first to identify novel RP susceptibility gene *SP‐D*. Three tagSNPs (rs1998374, rs911887, rs2255326) were identified as significantly associated with RP risk in the lung cancer patients treated with thoracic radiotherapy. The findings of our study may be useful in the development of genetic testing for the prediction of RP.

## CONFLICT OF INTEREST

None declared.

## AUTHOR CONTRIBUTIONS

Study conception and design: Hua Li, Li Xu, Junhong Jiang, Zhen‐zhou Yang, Bangxian Tan. Radiotherapy and RP diagnosis: Ling Zhang, Zhihui Li, Jing Xian, Chaoyang Jiang, Yong Diao, Xiaomei Su, Hongyu Xu, Tao Zhang, Zhen‐zhou Yang, Follow‐up of RP patients: Li Xu, Junhong Jiang, Zhihui Li, Jing Xian, Chaoyang Jiang, Yong Diao, Zhen‐zhou Yang, Yue Zhang, Data collection: Li Xu, Junhong Jiang, Hua Li, Yong Diao, Chaoyang Jiang. Data analysis: Yunming Li, Li Xu, Junhong Jiang, Hua Li, Bangxian Tan.
