# Supplementary material for: Genetic variants of SP‐D confer susceptibility to radiation pneumonitis in lung cancer patients undergoing thoracic radiation therapy
Source: Cancer Med. 2019 Mar 21;8(5):2599–611. doi: 10.1002/cam4.2088 (PMC6536953; doi:10.1002/cam4.2088)
Supplement: Supplementary file 1 [file CAM4-8-2599-s001.docx]

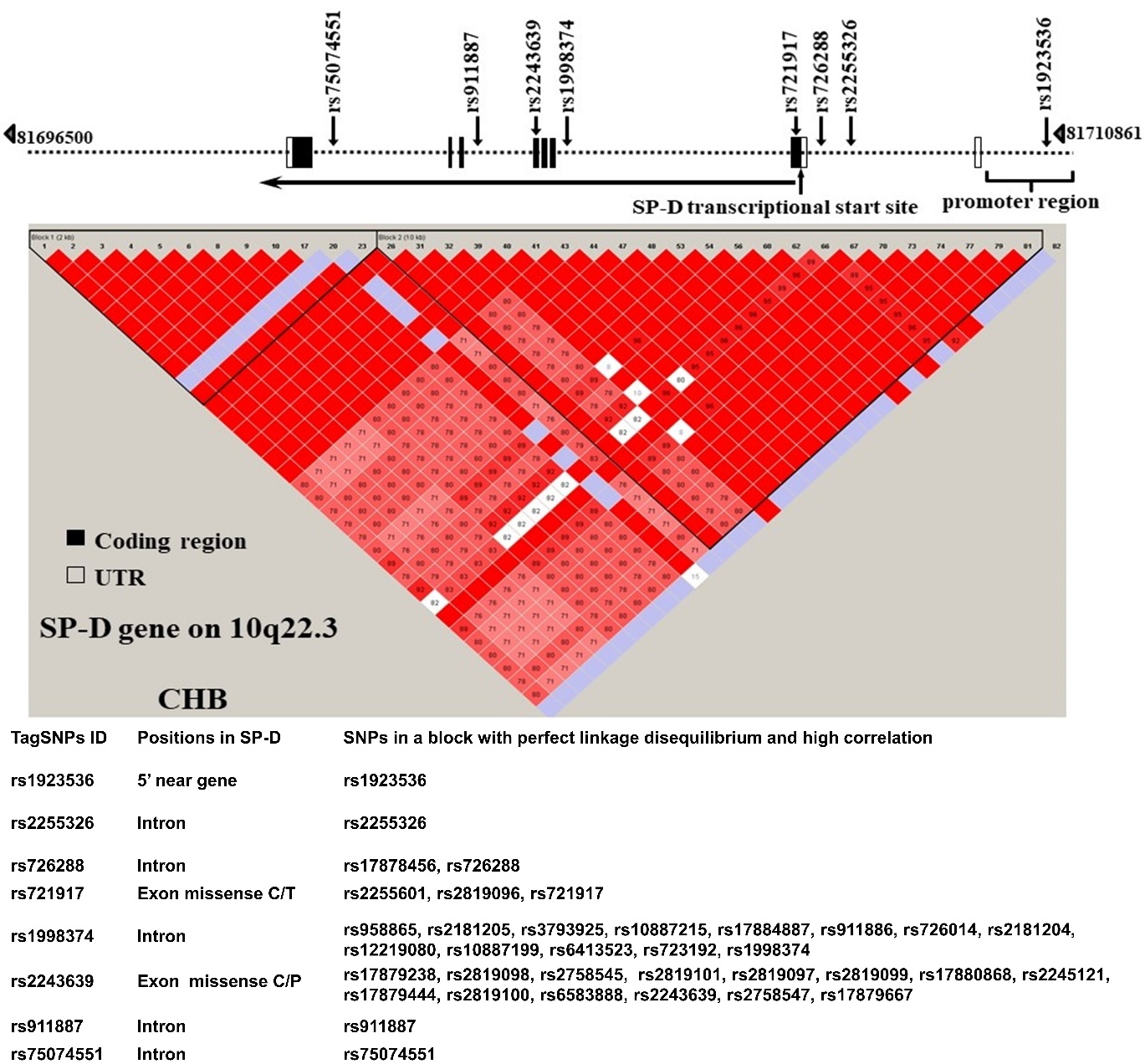


Supplementary Figure*.* Linkage disequilibrium of the *SP-D* in Han Chinese in Beijing, China (CHB). A schematic view of pairwise linkage disequilibrium between common single-nucleotide polymorphisms (SNPs) with minor allele frequency (MAF) >0.1 in a 14.36-kb region on chromosome 10q22.3 containing SP-D was shown. Haplotype blocks were defined by confidence intervals with Haploview 4.1. Red represents very high correlation (r^2^=1), pink represents medium correlation (0<r^2^>1), and white represents no correlation (r^2^ = 0) between 2 adjacent SNPs. The black rectangles in the SP-D represent exons, and the dotted line represents introns. The transcription direction of SP-D is reverse as shown by the long arrow. The locations of tagSNPs selected were shown as short arrows. Each tagSNP had the perfect linkage disequilibrium and high correlation with the SNPs in the same block (r^2^ > 0.8) as shown in the table. The 8 tagSNPs captured 100% of 36 alleles with MAF > 0.1 in *SP-D* with a mean r^2^ of 0.959.
